# Identification LEF1 as a Potential Novel Biomarker for Abdominal Aortic Aneurysms Based on Comprehensive Bioinformatics Analysis

**DOI:** 10.1111/jcmm.70921

**Published:** 2025-11-09

**Authors:** Pan He, Xinyi Liu, Hanshen Luo, Yuehang Yang, Jiong Guo

**Affiliations:** ^1^ Henan Provincial Chest Hospital Chest Hospital of Zhengzhou University Zhengzhou Henan Province China; ^2^ Department of Cardiovascular Surgery, Beijing Aortic Disease Center Beijing Anzhen Hospital of Capital Medical University Beijing China; ^3^ Department of Cardiovascular Surgery Union Hospital, Tongji Medical College, Huazhong University of Science and Technology Wuhan Hubei China

**Keywords:** abdominal aortic aneurysm, comprehensive bioinformatics analysis, key genes, LEF1, RNA‐seq analysis, ScRNA‐seq analysis

## Abstract

Abdominal aortic aneurysm (AAA), a life‐threatening cardiovascular disorder, necessitates the identification of novel molecular biomarkers to facilitate early diagnosis and precision therapeutic interventions. In this study, we employed an integrative bioinformatics strategy to systematically identify and characterise a potential biomarker for AAA. By reanalyzing GEO datasets and applying Lasso regression, we identified 10 candidate genes, whose intersection with known AAA‐associated genes pinpointed LEF1 as a pivotal regulator. Single‐cell transcriptomic analysis further demonstrated that LEF1 is predominantly expressed in aortic wall‐resident T cells, suggesting a spatially restricted regulatory role. Functional enrichment analysis highlighted significant associations with MHC class II protein complex binding and ribosomal structural integrity, implicating LEF1 in immune and translational regulation. Immunohistochemical analysis demonstrated significantly elevated expression of CD3, CD4 and CD8 markers in AAA tissues compared to controls. Flow cytometry and immunofluorescence analyses confirmed LEF1 co‐localisation with both CD8^+^ effector T cells and CD4^+^ memory T cells, with significantly enhanced LEF1 expression in AAA specimens versus controls. Overall, our study systematically discovered an important hub gene LEF1, which may serve as a biomarker for AAA.

## 1 Introduction

Abdominal aortic aneurysms (AAA), a significant cause of death in developed countries, are defined as a diameter ≥ 30 mm or an increase of 50% beyond the normal aortic diameter, with the primary characteristic being progressive aortic dilation [1]. If timely surgical intervention for AAA is not performed, the rupture mortality rate exceeds 80%, with 65% of patients showing no obvious symptoms before arterial rupture leads to death; therefore, surgical intervention has been shown to be an effective treatment that can prevent the risk of arterial rupture and death [2]. Ultrasound is the most cost‐effective imaging technique for detecting AAA, demonstrating extremely high sensitivity for aneurysms with a diameter > 30 mm [3]. Once an AAA with a diameter > 5.5 cm is detected by ultrasound, it should be treated with endovascular repair or open surgery as soon as possible [4]. However, AAAs are often found to be < 5.5 cm, so early and timely intervention for AAA has great potential clinical value [5].

Chronic inflammation plays a major role in the onset and progression of AAA. Inflammatory mediators can lead to the destruction of the aortic media and dysfunction and apoptosis of vascular smooth muscle cells (VSMCs). Additionally, a large number of innate immune cells (such as neutrophils and macrophages) and adaptive immune cells (such as T lymphocytes and B lymphocytes) infiltrate the aortic wall, resulting in the loss of elasticity in the vessel wall [6]. Among them, T cells serve as a major cellular component in AAA and can release pro‐inflammatory cytokines such as TNF‐α, IL‐5, IL‐6 and IFN‐γ, which promote tissue inflammation [7]. The functional deficiency of CD4^+^ CD25^+^ T regulatory cells is also involved in the pathogenesis of AAA [8]. Additionally, increasing research focuses on pharmacological interventions for AAA with diameters < 5.5 cm. However, evidence from studies on medications like beta‐blockers, ACE inhibitors and statins is limited, with only some antibiotics showing slight reductions in AAA expansion rates. Currently, there is still a lack of effective drugs to slow AAA progression in clinical [9]. Therefore, investigating the mechanisms of AAA at the molecular and cellular levels is a crucial potential foundation for developing effective treatments for the disease.

In this study, we downloaded four datasets from the Gene Expression Omnibus (GEO) database and obtained significant biomarkers by LASSO logistic regression model based on screened DEGs. Then, we performed an intersection between these key biomarkers and AAA‐associated genes to obtain LEF1. We then assessed the expression of LEF1 in AAA cell clusters using single‐cell RNA sequencing (scRNA‐seq). Finally, we successfully validated the expression of LEF1 in human samples and mouse models using IF and flow cytometry.

## 2 Materials and Methods

### 2.1 Data Collection

We obtained gene expression profiling data (GSE47472, GSE57691, GSE7084 and GSE166676) from the GEO database (www.ncbi.nlm.nih.gov/geo/). GSE47472, GSE57691 and GSE7084 contain microarray data. GSE166676 contains scRNA‐seq data. The GSE57691 and GSE47472 platforms are derived from GPL10558 and contain 63 AAA samples and 18 normal tissue samples in both datasets [10, 11]. GSE7084 is used for further internal dataset validation [12]. Four disease samples and two control samples are included in the scRNA‐seq dataset GSE166676, which is based on the GPL24676 platform [13]. The characteristics of the four datasets are shown in (Table S1). We downloaded information about 2501 genes related to ‘Abdominal aortic aneurysm’ from GeneCards (https://www.genecards.org/).

### 2.2 Screening and Integration of DEGs

The affy package in R was utilised for the pre‐processing of the original data, encompassing background correction and normalisation [14]. The datasets from GSE51472 and GSE12644 were integrated using the sva package in R. The DEGs were screened using the R package limma, applying cutoffs of *p* < 0.05 and |log fold change (FC)| > 1 [15]. The DEG volcano map was drawn using the ggplot2 R package (https://ggplot2.tidyverse.org).

### 2.3 Screening and Validation of the Hub Gene of AAA

Based on the identified DEGs, we utilised LASSO regression to screen for biomarkers associated with AAA. LASSO logistic regression was performed using the glmnet R package [16]. Lastly, we utilised the ggplot2 R package to create a Venn plot, enabling us to identify the pivotal gene associated with AAA.

### 2.4 Single‐Cell Sequencing Data Preprocessing and Cell Type Annotation in GSE166676

The scRNA‐seq data derived from the GSE166676 dataset underwent preprocessing using the Seurat R package [17]. We filtered cells based on their expression of 200 to 2500 genes and < 5% mitochondrial genes. The top 2000 hypervariable genes were identified using the ‘FindVariableFeatures’ function in Seurat, which was followed by running PCA analysis to determine 14 principal components (PCs). Subsequently, a ‘tSNE’ diagram was generated to visualise the clustering of all cell populations.

To identify differentially expressed genes (DEGs) within each cell cluster, we employed the ‘FindMarkers’ function in Seurat. To select marker genes for each cell cluster, we considered known roles of these genes from the database ‘HumanPrimaryCellAtlasData’, as well as their spatial relationships within the tSNE plot. Notably, there were no discrepancies observed between our identified marker gene list and those reported in the original research study [13].

### 2.5 Pseudotime Trajectory Analysis

We extracted all cell objects annotated as T cells and performed pseudotime analysis using the Monocle R package [18]. First, we reduced the dimensionality of the data using the ‘DDRTree’ function in Monocle. Then, we utilised the ‘reduceDimension’, ‘orderCells’ and ‘plot cell trajectory’ functions to identify distinct cell differentiation states and visualise their corresponding trajectories.

### 2.6 Functional and Pathway Enrichment Analysis

We conducted Gene Ontology (GO) functional analysis and Kyoto Encyclopedia of Genes and Genomes (KEGG) pathway enrichment on the relevant DEGs using the clusterProfiler R package [19]. A significance level of *p* < 0.05 was set to determine the statistically significant results.

### 2.7 Estimation of Immune Cell Infiltration

We quantified the degree of immune cell infiltration of the combined transcriptome data using a deconvolution CIBERSORT algorithm based on the CIBERSORT R package (http://CIBERSORT.stanford.edu/) with parameter ‘perm’ set to 1000 and a cut‐off of *p* < 0.05. The proportion of each type of immune cell in the sample is calculated and shown with a bar chart; a heatmap of immune cells was created using the pheatmap R package and the corrplot R package was used to visualise the correlation between different infiltrating immune cells.

### 2.8 Human Tissue Collection

Human tissue collection was conducted as previously described [20]. All protocols using human specimens were approved by the Human Research Ethics Committees of Union Hospital, Tongji Medical College, Huazhong University of Science and Technology ([2024] IACUC Number: 0001), and informed consent was obtained from patients or their family members. The AAA wall tissue samples were collected from the patients who underwent surgical repair of AAA, while the normal aortic tissues were the abandoned aortae from heart transplantation. These human specimens were stored in paraformaldehyde in time after aorta excision.

### 2.9 Flow Cytometry

Briefly, mouse aortic tissue was digested into a single‐cell suspension using RPMI‐1640 medium containing 2 mg/mL collagenase I and 0.2 mg/mL DNase. Fresh tissue was rinsed with PBS to remove blood, platelets and then cut into 2 mm pieces with sterile scissors. The tissue fragments are placed in the digestion solution and incubated for 60 min at 37°C on a shaking platform. After digestion, the solution is filtered through a 100 μm cell strainer to remove undigested fibres. The filtrate is centrifuged and the precipitate is resuspended in PBS and centrifuged again to remove the residual digestive solution. The final precipitate (100–200 μL) was used for flow cytometry staining. Cell viability was assessed in flow cytometry using the Zombie Aqua Cell Viability Kit (Biolegend, Catalogue # 423101) followed by membrane permeabilisation fixation. Fc receptor blocking was performed using FC‐block to reduce non‐specific antibody binding. Cells are incubated with fluorochrome‐conjugated antibodies against specific surface markers to label the relevant cell populations according to the manufacturer's instructions.

### 2.10 AAA Animal Model Induced by Ang II

The AAA animal model was performed as previously described [20]. ApoE‐knockout (ApoE^−/−^) male mice and C57BL/6 male mice were procured and housed in a meticulously maintained, pathogen‐free barrier facility. To induce the development of AAA, male mice were subjected to the infusion of Ang II (1000 ng/kg/min) or normal saline via an implanted osmotic pump over a period of 7 days. After 4 weeks of treatment, aortic diameters were determined by transthoracic echocardiography using an 18–38 MHZ phased‐array probe (MS400) connected to a Vevo 2100 Imaging system under 2.5% isoflurane anaesthesia. Subsequently, the mice were euthanised, and comprehensive histological and molecular analyses were conducted. Abdominal aortic tissues were obtained from both AAA‐induced mice and normal mice that had undergone the previously described surgical procedures. These tissues were either preserved at −80°C or subjected to formalin fixation and subsequently embedded in wax blocks for further examination. Ethical approval for all animal studies was obtained from the Institutional Animal Care and Use Committee of Huazhong University of Science and Technology ([2024] IACUC Number: 4389). The experiments adhered to the guidelines outlined in the ‘Guide for the Care and Use of Laboratory Animals’ (National Institutes of Health publication no. 85–23, 1996). Stringent adherence to the relevant guidelines and regulations was ensured throughout the course of the experiments.

### 2.11 Histology and Immunofluorescence Staining

The freshly collected tissue from the mouse abdominal aortic wall was preserved using a 4% paraformaldehyde solution. Subsequently, the tissue was embedded in paraffin and sectioned into slices with a thickness of 5 μm. These sections were then subjected to various staining techniques, including haematoxylin–eosin (HE), Verhoeff–Van Gieson (EVG) and Masson's trichrome staining. The stained sections were examined using a light microscope. For immunofluorescence staining, firstly, they were heated in a water bath, followed by deparaffinisation. Next, antigen retrieval was performed, and the sections were cooled to room temperature before being fixed and blocked. Antibodies against LEF1 (14972‐1‐AP; Proteintech, 1:600) were then applied for staining. Finally, the specimens were observed under a confocal laser scanning microscope (Olympus, Tokyo, Japan).

### 2.12 Statistical Analyses

In this study, all statistical analysis of data and graphing was completed by GraphPad Prism 8.0 software. For differences between two independent samples, the student t‐test was used to analyse the data. Comparisons of data between multiple sample groups were statistically calculated by one‐way or two‐way analysis of variance (ANOVA). All statistical results are reported as mean ± standard error (Mean ± SEM). When the *p*‐value was < 0.05, we considered a statistically significant difference between the results.

## 3 Result

### 3.1 Screening of DEGs and Identification of Hub Genes

**FIGURE 1:**
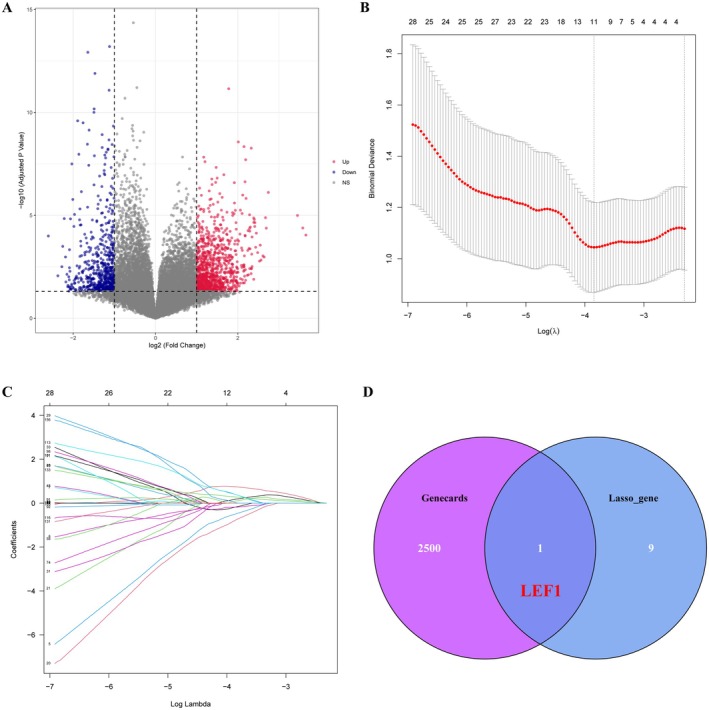
Screening of DEGs and identification of hub genes. (A) Volcano plots illustrating the differential expression of genes (DEGs) derived from the combined GSE47472 and GSE57691 datasets. Upregulated genes are represented by red dots, downregulated genes by blue dots and genes with no significant difference in expression by grey dots. (B) The least absolute shrinkage and selection operator (LASSO) model was employed for refined feature selection. LASSO regression was utilised to narrow down the DEGs, resulting in the identification of 10 potential markers for AAA. The coordinates on the plot represent coefficient values, with the lower abscissa indicating logarithm (*λ*) values and the upper abscissa representing the current number of nonzero coefficients in the model. (C) The LASSO Logit model algorithm filters diagnostic markers and presents lambda values visually. (D) A Venn diagram illustrates the intersection between biomarker genes obtained through LASSO regression analysis and AAA‐related genes listed in GeneCards. Ultimately, LEF1 gene emerged as a hub gene based on this analysis.

The study flow chart is shown in Figure 1. The datasets GSE47472 and GSE57691 from the GEO database were acquired and downloaded. After obtaining the data, the two datasets were merged and the microarray data was subjected to normalisation. Subsequently, utilising the limma R program, DEGs of the combined dataset were filtered using a threshold of |log FC| ≥ 1 and *p* < 0.05. After removing batch variation and standardising the data, a total of 1601 DEGs were identified in the combined dataset. Among these, 982 genes were found to be up‐regulated while 619 genes exhibited down‐regulation (Figure 1A; Data S1). Afterwards, we utilised the LASSO logistic regression model to identify 10 significant biomarkers from the screened DEGs. These biomarkers include genes such as STAC2, CCDC71, ALDH1L1, PNISR, ZNF575, BTC, WDR82, NPAS2, LRRC57 and LEF1 (Figure 1B,C). Subsequently, in order to obtain a pivotal gene related to AAA, we performed an intersection between these 10 key biomarkers obtained through LASSO logistic regression and AAA‐associated genes in GeneCards. As a result of this analysis, the key gene identified was LEF1 (Figure 1D).

### 3.2 Single‐Cell Sequencing Data Analysis

**FIGURE 2:**
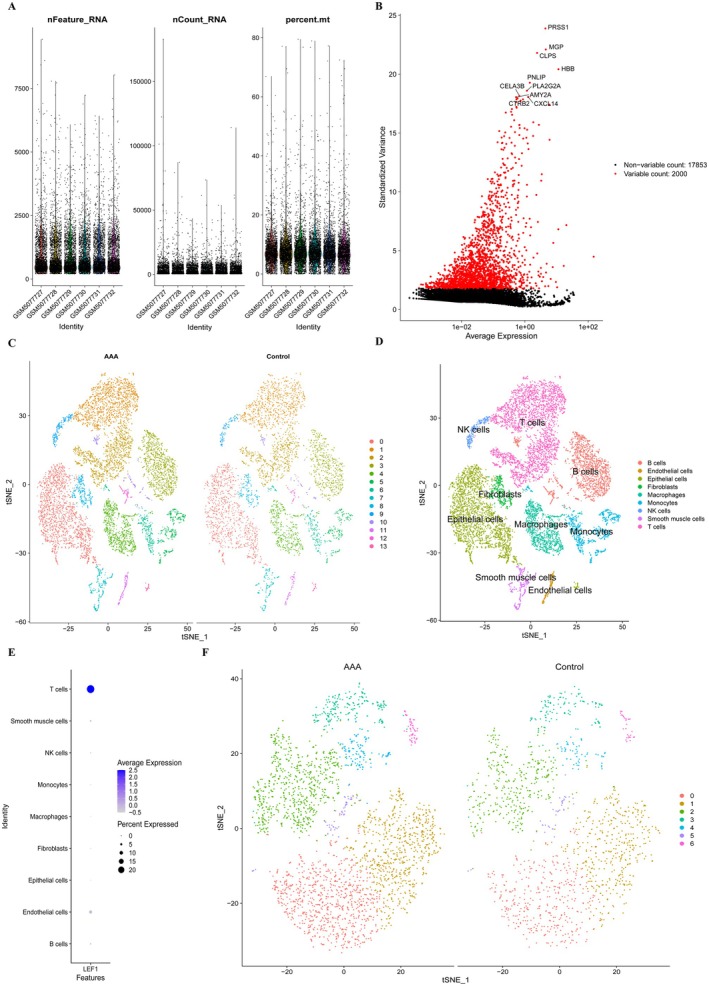
Single‐cell sequencing analysis in the GSE166676 dataset. (A) Three criteria were used to ensure cell sample quality: RNA count, gene count and mitochondrial gene percentage. (B) The top 10 highly variable genes are highlighted in red among the 2000 most highly variable genes. (C) Dimensionality reduction and clustering analysis were done on the cells in the dataset, and the ‘tSNE’ diagram depicts the grouping of the AAA and control groups. (D) Annotation of the clustered cells, including T cells, smooth muscle cells, fibroblasts, macrophages, NK cells, endothelial cells, monocytes, Epithelial cells and B cells. (E) Expression of LEF1 in cell clusters. (F) The T cells in the dataset are extracted for dimensionality reduction and cluster analysis, and the cell cluster clustering of T cells in the AAA and control groups is displayed in the ‘tSNE’ diagram.

The GSE166676 scRNA‐seq dataset was downloaded from the GEO database to investigate the expression of LEF1 in different cell groups within the aorta wall of AAA patients. To ensure sample quality, we conducted quality control measures by eliminating cells and genes of poor quality (Figure 2A). The top 2000 highly variable genes were identified, with the first 10 genes labelled accordingly (Figure 2B). Following dataset normalisation and principal component analysis, cells were classified into 14 clusters (Figure 2C). Subsequently, cell annotations were assigned based on cluster‐specific gene expression (Figure 2D), and a comprehensive list of marker genes can be found in (Data S2). As illustrated in (Figure 2E), LEF1 exhibits predominant expression within T cells residing in the aorta wall. Consequently, we isolated the T cell population from the dataset, divided it into seven cell clusters through dimensionality reduction and clustering (Figure 2F).

### 3.3 Functional Enrichment and Pseudotemporal Analysis

**FIGURE 3:**
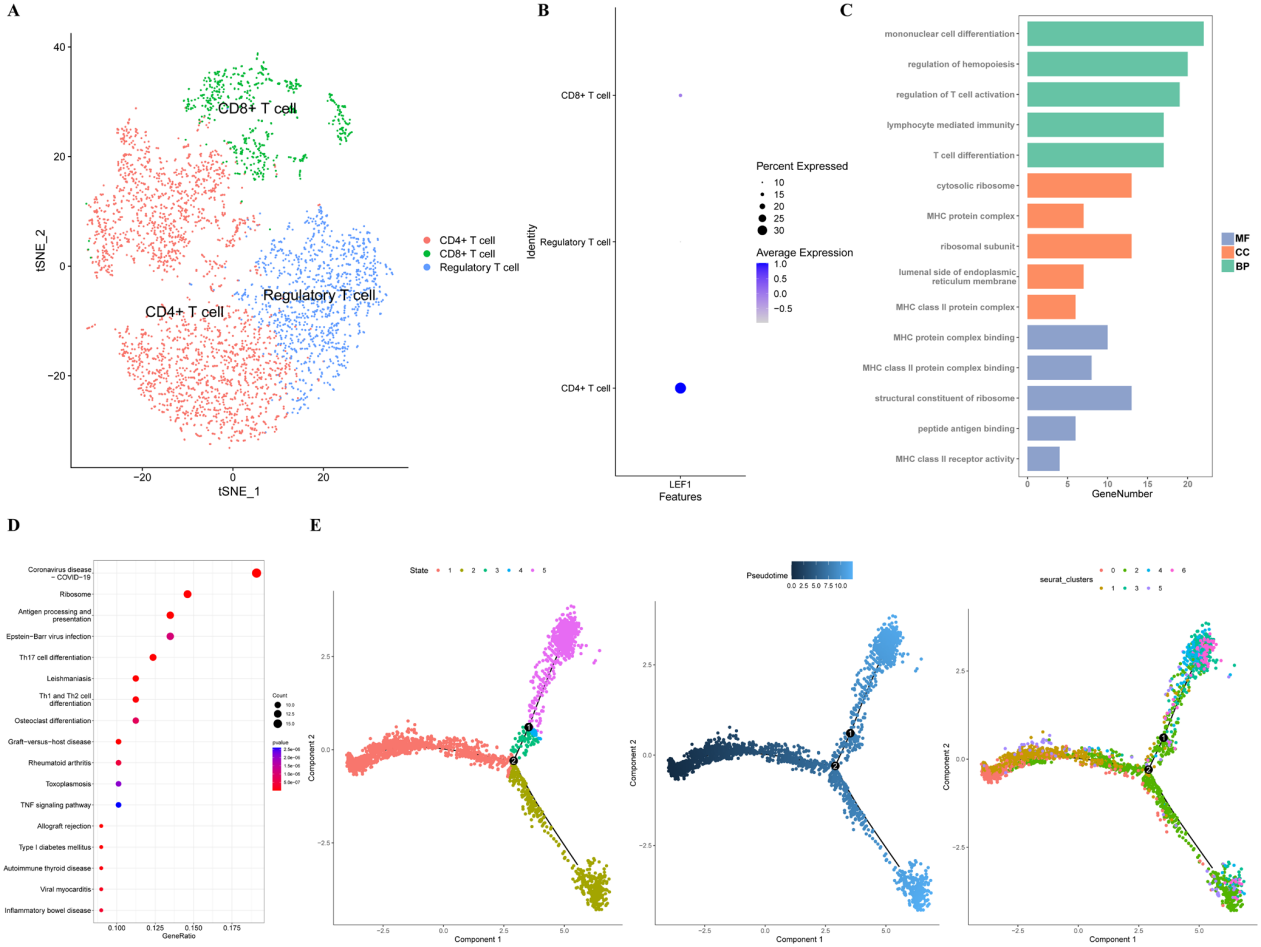
Functional enrichment and pseudotemporal analysis of T cells in the GSE166676 dataset. (A) The annotated T cell cluster group contains CD8^+^ T cells, CD4^+^ T cells and Regulatory T cells. (B) LEF1 expression in T cell clusters. (C) Gene Ontology (GO) enrichment analysis of DEGs between LEF1‐significant T cell clusters and other LEF1‐less cell clusters. (D) Kyoto Encyclopedia of Genes and Genomes (KEGG) enrichment analysis based on DEGs between LEF1‐significant T cell clusters and other LEF1‐less cell clusters. (E) Pseudotime analysis of T cells is depicted in the illustration, showcasing five distinct differentiation states. The images highlight the aggregation of seven cell clusters and the variations in cell differentiation over time.

**FIGURE 4:**
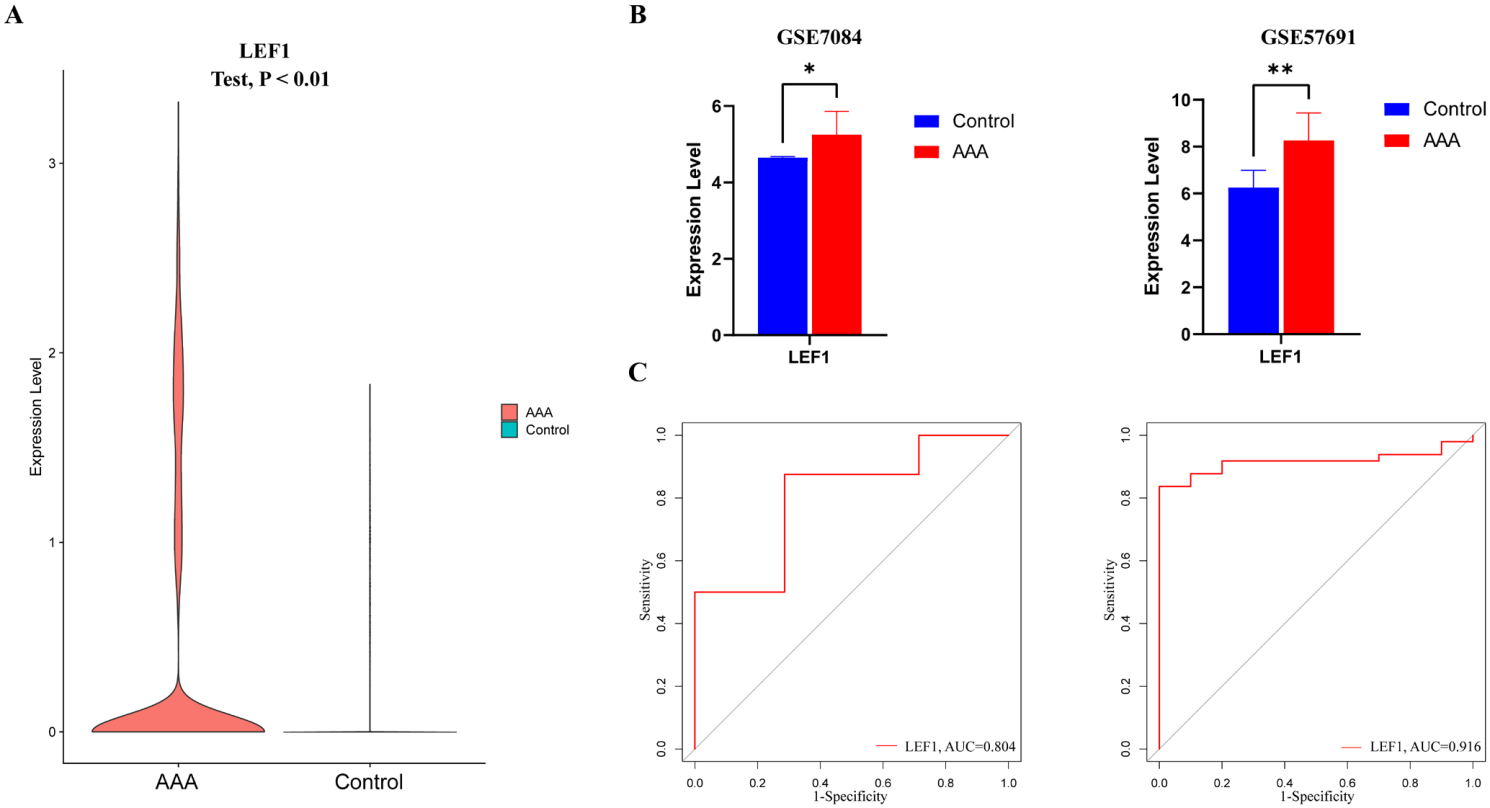
Validation of LEF1 in datasets from the GEO database. (A) LEF1 expression levels in the single‐cell dataset GSE166676. (B) LEF1 expression level in the two datasets GSE7084 and GSE57691. (C) LEF1 receiver operating characteristic (ROC) curves in the two datasets GSE7084 and GSE57691 demonstrate their diagnostic value. *p < 0.05, **p < 0.01 when two groups were compared as indicated or compared to the corresponding control.

By using marker genes, we separate the aforementioned seven T cell clusters into CD8^+^ T cells, CD4^+^ T cells and Regulatory T cells (Figure 3A). In (Data S3), a list of the specific T cell grouping marker genes is provided. As shown, the expression of LEF1 is significantly higher in CD4^+^ T cells compared to CD8^+^ T cells (Figure 3B). To delve into the potential function of LEF1 in T cells residing within the aorta wall, we screened LEF1‐significant cell clusters and other LEF1‐less cell clusters for differential genes, and performed GO and KEGG enrichment analyses. The detailed DEGs that resulted are displayed in (Data S3). The outcomes of the GO analysis included biological processes, cellular components and molecular functions (Figure 3C). The set of identified genes is enriched mostly in biological processes such as MHC protein complex binding and structural constituent of ribosome. The molecular functions mainly included regulation of hemopoiesis and regulation of T cell activation. Cellular components included cytosolic ribosome and MHC protein complex. Antigen processing and presentation, the TNF signalling pathway, Th17 cell differentiation and Th1 and Th2 cell differentiation were among the enriched pathways in the KEGG pathway enrichment analysis (Figure 3D). Finally, we performed pseudo‐temporal analysis to predict cell trajectories of T cells (Figure 4E). In the pseudotime analysis, T cells are divided into five different differentiation states. It is worth noting that cell clusters 0, 1, 5 are in the early stages of differentiation, cell clusters 2, 3 are in the intermediate stage, and cell clusters 4, 6 are in the terminal stage of differentiation.

### 3.4 Validation of LEF1 in GEO Datasets

As shown in (Figure 4A), the expression of LEF1 in AAA in single‐cell data was significantly upregulated compared with normal samples (*p* value < 0.01). Next, we perform internal validation of the GEO dataset through GSE7084 and GSE57691. Compared with the control group, the expression of LEF1 was significantly upregulated in the disease group (Figure 4B), and the area under the curve (AUCs) of LEF1 in both datasets was > 0.8, indicating that LEF1 can be used as a potential biomarker for the diagnosis of AAA patients (Figure 4C).

### 3.5 Immune Cell Infiltration Results

**FIGURE 5:**
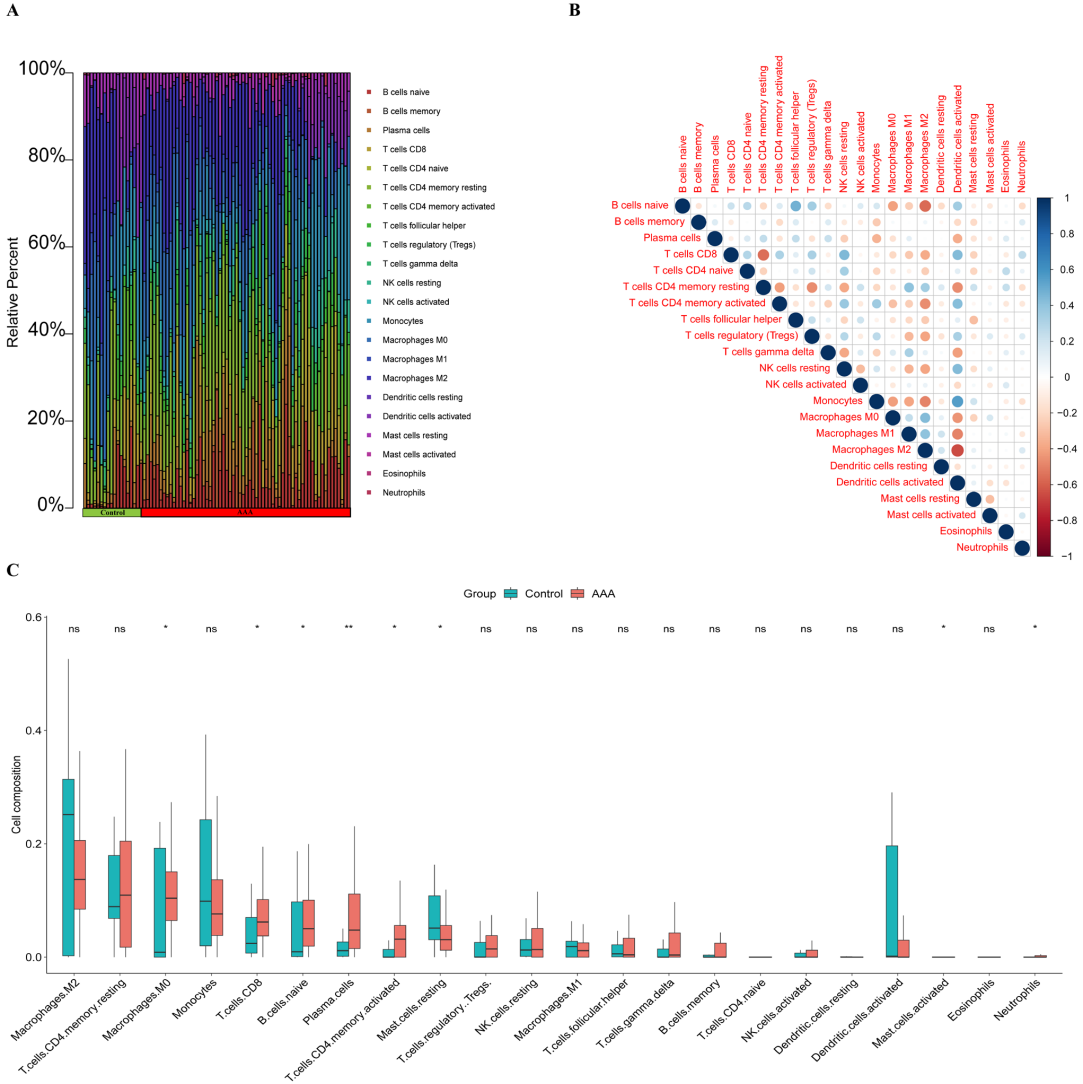
Immune infiltration analysis. (A) Immune cell infiltration ratio of abdominal aortic aneurysm (AAA) and control group. (B) Correlation analysis of different immune cells. Dark red indicates strong negative correlation, dark blue indicates strongest positive correlation. (C) Differences in the infiltration of various immune cells in the AAA and control groups. *p < 0.05, **p < 0.01 when two groups were compared as indicated or compared with the corresponding control. Ns indicates non‐significant.

The above bioinformatics analysis proves that LEF1 is closely related to immune function. To further explore this relationship, we employed the CIBERSORT tool to analyse immune cell infiltration using the uploaded combined transcriptome dataset. As depicted in (Figure 5A), macrophages and T cells emerged as the predominant immune cells within the aortic wall of AAA patients. Subsequently, we conducted a correlation analysis on these immune cells (Figure 5B), with shades of dark blue indicating robust positive correlations and deep red hues signifying pronounced negative correlations. We discovered a significant negative correlation between T cells CD4 memory resting and T cells CD8. Likewise, macrophages M2 exhibited a significant negative correlation with B cells naive. On the other hand, we found a notable positive correlation between T cells follicular helper and B cells naive. Simultaneously, (Figure 5C) illustrates a substantial increase in the infiltration of T cells CD8, T cells CD4 memory activated and macrophages M0 within the aortic wall of AAA patients.

### 3.6 Validation of T Cell Activation and Infiltration Within the Aortic Wall of AAA

**FIGURE 6:**
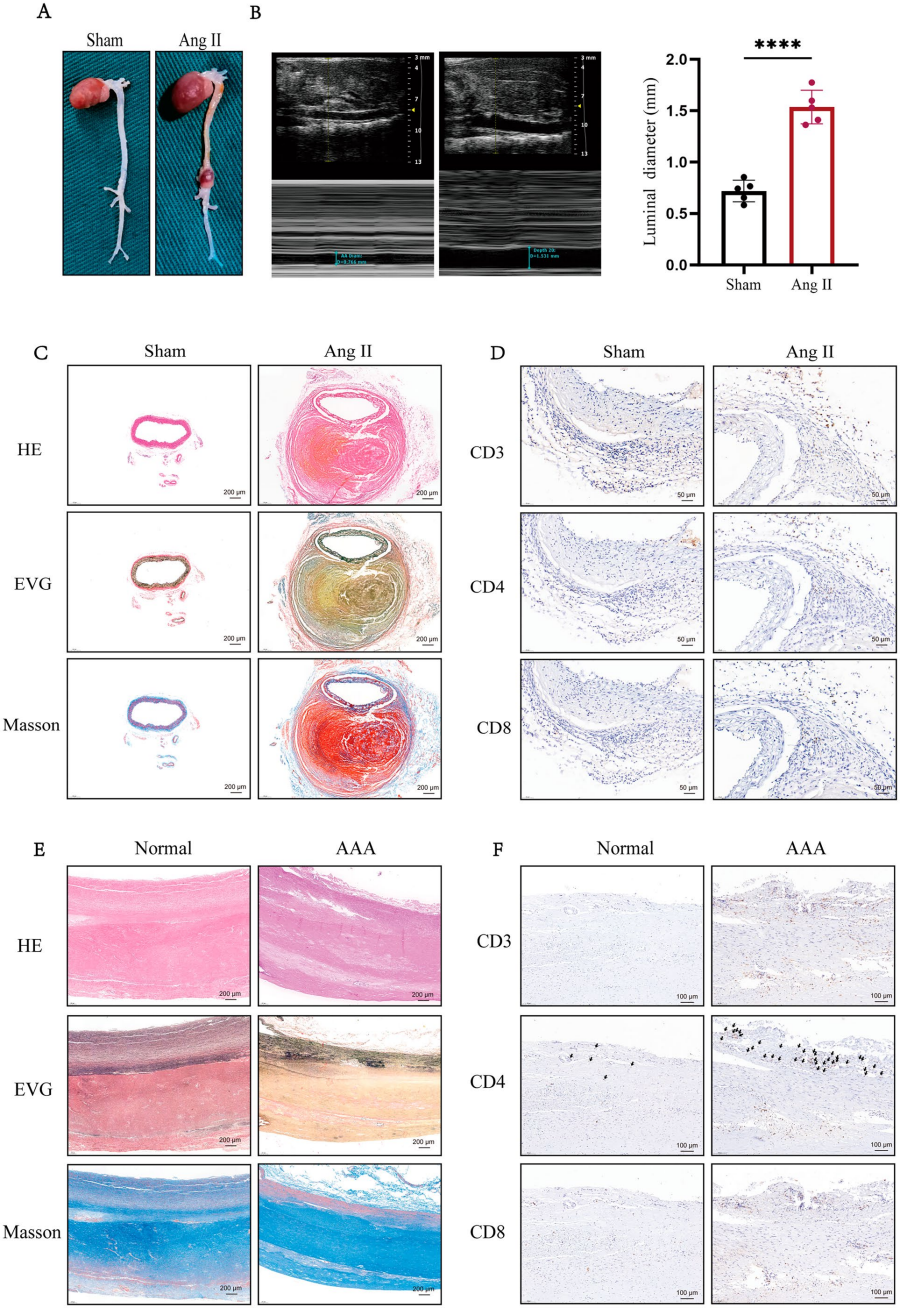
The validation of Immune infiltration in AAA. (A) Representative photographs showing macroscopic features of AAA induced by Ang II. (B) Representative ultrasound image of aortic dimension in two groups and quantification analysis (*n* = 5). (C) Representative images of HE, EVG and Masson staining of both sham and Ang II‐induction mouse aortic dissection aneurysm. (D) Representative images showing immunohistochemistry staining of CD3, CD4 and CD8 in the mouse aortae tissue from indicated groups. (E) HE, EVG and Masson staining showing the features of the aortic wall in the normal and AAA patients. (F) Immunohistochemistry staining showing the differences of CD3, CD4 and CD8 between normal and AAA patient's samples. ****p < 0.0001 when two groups were compared as indicated or compared with the corresponding control. ns indicates non‐significant.

To verify the T cell activation and infiltration within the aortic wall of AAA, we constructed a mouse aortic dissection and aneurysm model using Ang II induction. As shown in (Figure 6A), after 4 weeks, the Ang II‐induced mice had a significant aortic aneurysm formation, and the results of vascular Doppler ultrasound revealed a substantial increase in aortic diameter in the Ang II group of mice compared to the control group (Figure 6B). In addition, HE staining significantly showed aortic dissection formation in the Ang II group, while EVG and Masson staining verified marked fragmentation and disorganisation of elastic fibres in the aortic wall of the aortic aneurysm (Figure 6C). Immunohistochemical (IHC) staining confirmed that compared with the control group, T cell markers CD3, CD4 and CD8 were higher in the aortae of mice treated with Ang II (Figure 6D). Moreover, in order to validate the above conclusions, we collected normal and AAA patient aortic tissues. The results of HE staining, EVG and Masson staining revealed significant thickening of the intima and smooth muscle cell disorganisation, accompanied by a discernible decrease in elastin and collagen fibre density (Figure 6E). Furthermore, IHC staining of the aortic wall was performed, and a higher proportion of CD3, CD4 and CD8 positivity was observed than in the normal group, which was consistent with the mouse tissue results (Figure 6F). Collectively, these observations suggest massive T cell activation and infiltration within the aortic wall of AAA.

### 3.7 Validation of LEF1 in Ang II‐Induced Mouse Aortic Dissection Aneurysm Models

**FIGURE 7:**
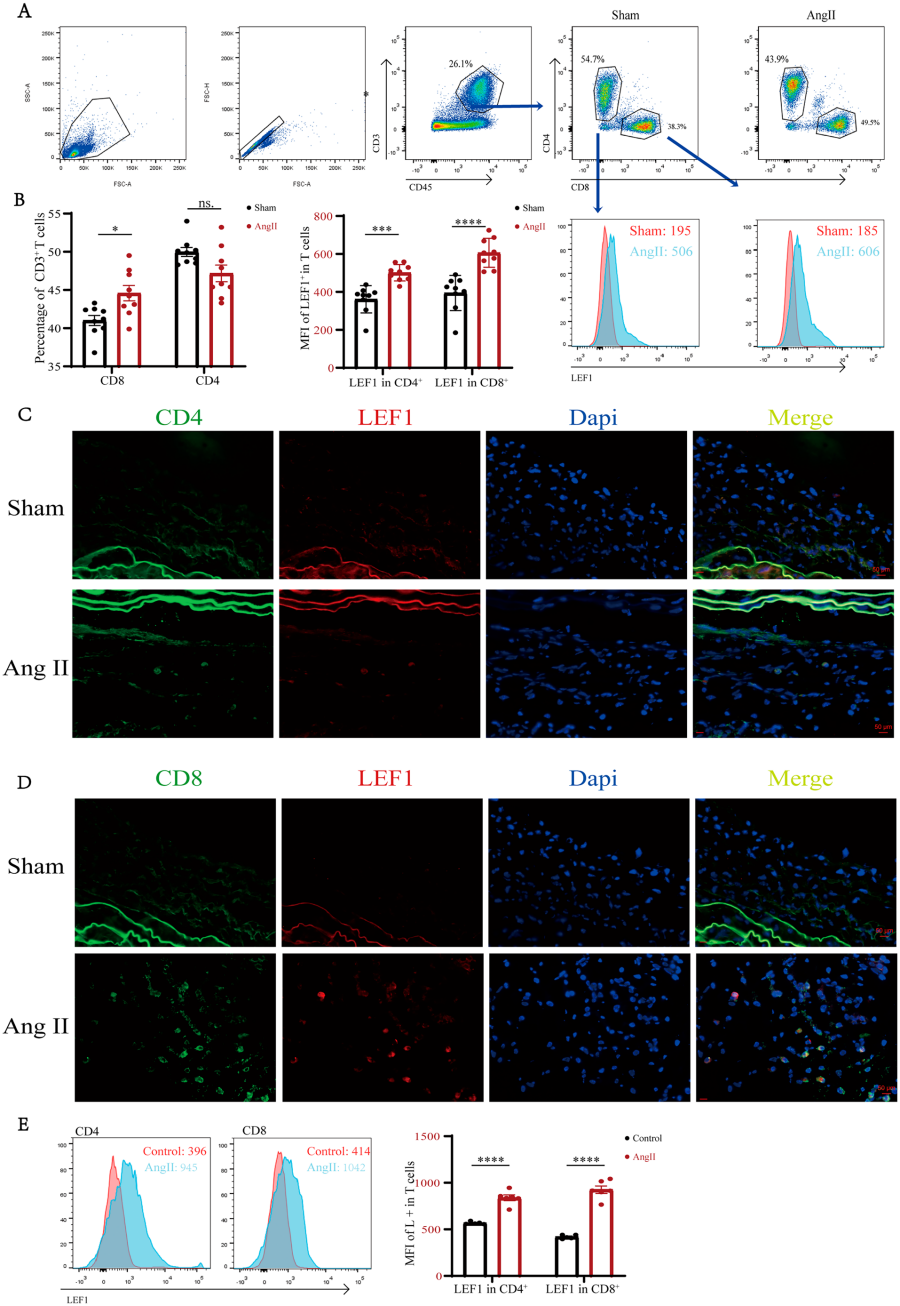
The validation of LEF1 expression in AAA. (A) The LEF1 expression levels in different groups of T cells CD8 and T cells CD4 memory were detected by flow cytometry. (B) Quantitative analysis of the proportion of T cells CD3 among T cells CD8 and T cells CD4 memory (*n* = 9). Quantitative analysis of mean fluorescence intensity of LEF1 in T cells CD8 and T cells CD4 memory (*n* = 9). (C) IF staining of CD4 and LEF1 in mouse aorta tissue for indicated treatments. (D) IF staining of CD8 and LEF1 in mouse aorta tissue for indicated treatments. (E) The LEF1 expression levels in different groups of T cells CD8 and T cells CD4 memory were detected by flow cytometry and quantitative analysis of mean fluorescence intensity of LEF1 in T cells CD8 and T cells CD4 memory (*n* = 6). *p < 0.05, ***p < 0.001, ****p < 0.0001 when two groups were compared as indicated or compared with the corresponding control. ns indicates non‐significant.

To evaluate the crosstalk between T cells and LEF1 in sham and Ang II mice, flow cytometry was then applied (Figure 7A). The results showed a higher proportion of T cell CD8 in the Ang II group relative to the sham group, while there was no significant difference in T cell CD4 memory (Figure 7B). Interestingly, the mean fluorescence intensity (MFI) of LEF1 was significantly higher in the Ang II group than in the sham group for both T cell CD8 and T cell CD4 memory (Figure 7B). Furthermore, immunofluorescence staining confirmed the co‐localisation of LEF1 with both T cells CD8 and T cells CD4 memory, and the expression of LEF1 was increased significantly in the Ang II group compared to the sham group (Figure 7C,D). In addition, the results of flow cytometry further showed the higher MFI of LEF1 in the Ang II group than in the control group (Figure 7E). Overall, the above experiments demonstrated that LEF1 was mainly enriched in activated CD8^+^ and CD4^+^ memory T cell subsets, and that LEF1 expression was positively correlated with the degree of T cell infiltration in AAA.

## 4 Discussion

AAA represents a life‐threatening cardiovascular disorder, pathologically defined by progressive segmental dilation of the abdominal aorta and associated with substantial mortality rates [21]. At present, surgical intervention remains the sole clinically validated approach for both the prevention and treatment of AAA. Despite extensive investigations into the pathophysiological mechanisms underpinning AAA in recent decades, the molecular and cellular drivers of its initiation and progression remain incompletely elucidated. In this study, we identified LEF1 as a novel biomarker for AAA through comprehensive bioinformatics and experimental validation. We first identified LEF1 as a central hub gene for AAA by DEG screening and LASSO regression; then, we found its predominant expression in T cells infiltrated within AAA lesions using scRNA‐seq, and functional enrichment linked LEF1 to immune pathways (e.g., T cell activation, MHC binding). Finally, these results were further validated in human AAA tissues and mouse models, where upregulation of LEF1 was associated with CD4^+^/CD8^+^ T cell infiltration. In conclusion, our data suggest that LEF1 serves as both a diagnostic marker and a mediator of immune‐driven AAA pathogenesis.

Chronic inflammation represents a hallmark pathological feature of aneurysmal aortic tissue. While the pathogenesis of AAA is multifactorial, structural compromise of the aortic wall facilitates transendothelial migration of inflammatory cells into the intimal and medial layers, where they exacerbate aneurysmal progression through the sustained secretion of pro‐inflammatory cytokines, reactive oxygen species (ROS) and extracellular matrix‐degrading mediators. The inflammatory infiltrate characteristic of aortic aneurysms comprises a diverse array of immune cell populations, including but not limited to macrophages, neutrophils, mast cells, natural killer (NK) cells, dendritic cells (DCs), as well as adaptive immune components such as B lymphocytes and T lymphocytes [22]. Among these immune effectors, T lymphocytes—classifiable into CD4^+^ T helper (Th) cells, cytotoxic CD8^+^ T cells and γδ T cell subsets—have been demonstrated to exert pivotal functions in AAA pathogenesis [23]. Notably, CD4^+^ Th cells constitute the predominant immunocyte population within the aneurysmal aortic wall microenvironment, where they orchestrate key pathological processes through cytokine‐mediated mechanisms [24]. Accumulating evidence from murine models has established that genetic ablation of CD4^+^ T cells confers protection against aneurysm development, mechanistically linked to attenuated interferon‐gamma (IFN‐γ) signalling pathways [25]. Furthermore, multiple interleukins signalling pathways have been implicated in AAA pathogenesis through their capacity to drive Th1 cell polarisation and clonal expansion, with CD4^+^ T lymphocytes serving as the principal cellular targets. Experimental evidence from established AAA models demonstrates that targeted inhibition of IL‐12 in elastase‐perfusion models and IL‐2 blockade in angiotensin II (Ang II)‐infusion models significantly attenuates disease progression, highlighting cytokine‐specific therapeutic potential [26, 27]. In contrast, the role of Th2 cells in AAA is under debate, even though it is widely recognised as having anti‐inflammatory effects. Emerging evidence suggests that Th2‐derived IL‐4 and IL‐5 paradoxically exacerbate AAA progression by simultaneously upregulating MMP‐2/9 expression and inducing VSMC apoptosis—two hallmark pathological features of aortic wall degeneration [28]. Notably, exogenous IL‐10 administration significantly attenuated aortic wall inflammation in rabbit AAA models through immunomodulatory reprogramming of Th1/Th2 cytokine balance, ultimately retarding aneurysmal progression as evidenced by reduced expansion rates and preserved medial integrity [29]. For CD8^+^ cells, widely recognised as exacerbating AAA progression, this pathogenic role was definitively established by Lin et al., who reported that genetic ablation of CD8^+^ T cells conferred significant protection against CaCl2‐induced AAA formation in murine models, as evidenced by reduced aortic dilation and preserved elastin integrity. The possible underlying mechanism for this is that CD8^+^ T cells regulate IFN‐γ‐dependent inflammatory responses and oxidative stress [30]. Moreover, significantly higher levels of CD8^+^ T cells were found in the serum of AAA patients compared with healthy subjects [31]. Consistent with these results, in our study, we found a large infiltration of T cells in the aortic wall using the uploaded combined transcriptome dataset. Furthermore, we confirmed this conclusion by staining CD4 and CD8 in the aortic wall of AAA patients as well as mouse AAA models, respectively. The above studies suggest that T cells play a significant role in the progression of AAA.

LEF1, a member of the T‐cell Factor (TCF)/LEF1 family of high‐mobility group transcription factors, which main function is to control the development and maturation of T cells [32]. In our study, we confirmed that LEF1 is predominantly expressed in T‐cell subsets in the aortic wall by scRNA‐seq dataset. In addition, as a key player of the Wnt/β‐catenin signalling pathway, LEF1 drives the expression of genes involved in tumorigenesis, cancer cell proliferation, stem cell maintenance, embryonic and organ development [33]. At present, a large number of studies have demonstrated that LEF1 is linked to many cardiovascular diseases through the Wnt/β‐catenin signalling pathway, such as hypertrophic cardiomyopathy, myocardial infarction and myocardial fibrosis [34, 35, 36]. In the context of AAA, research should primarily focus on the crosstalk between LEF1 and both VSMCs and endothelial cells. Previous studies have reported that Wnt3a activates β‐catenin signalling and upregulates the mRNA levels of AXIN2, LEF1, RUNX2 and BMP4 (early osteogenic genes) in HASMCs [37]. Another study has demonstrated that LEF1‐AS1 promotes phenotypic switching of VSMCs by regulating the Hippo/Yap signalling pathway [38]. Additionally, apoptosis in cardiac cells can be induced through activation of Wnt signalling [39]. The aforementioned studies demonstrate that LEF1 may contribute to the progression of AAA by regulating VSMC calcification, phenotypic switching, and apoptosis—processes widely recognised as pivotal in AAA pathogenesis [40]. Beyond its role in VSMCs, LEF1 may also engage in crosstalk with endothelial cells, thereby influencing the progression of aortic aneurysms. For example, silencing of endothelial vWF has been shown to reduce angiotensin II–induced endothelin‐1 expression [41], suggesting that a LEF1‐vWF‐ET‐1 axis might contribute to endothelial dysfunction in AAA. Moreover, the canonical Wnt/β‐catenin signalling pathway has been demonstrated to be closely associated with vascular endothelial cell dysfunction [42]. In our study, we identified LEF1 as a hub gene for AAA by bioinformatics analysis. Furthermore, we reanalysed single‐cell sequencing data and functionally enriched subpopulations of cells with high LEF1 expression, including T cell activation, which may be associated with the development of AAA. Moreover, we validated LEF1 as a potential biomarker for predicting AAA through the GEO database. We further verified by in vivo and in vitro experiments that the expression of LEF1 was significantly up‐regulated in the vascular wall of Ang II‐induced aortic aneurysms in mice and AAA patients, as well as in T cells infiltrated, suggesting that LEF1 has the potential to regulate AAA development through the modulation of T cells, and is highly likely to be a potential biomarker.

While our study demonstrates LEF1's association with T‐cell infiltration in AAA, its specific functional roles warrant further investigation. As a transcription factor in the Wnt/β‐catenin pathway, LEF1 is known to regulate T‐cell development and differentiation [32]. In AAA, LEF1 may promote pro‐inflammatory T‐cell polarisation (e.g., Th1/Th17) through transcriptional activation of cytokine genes (e.g., IFN‐γ, IL‐17) and modulation of T‐cell receptor signalling. This hypothesis is supported by our enrichment analyses showing LEF1‐high T cells are associated with Th17 differentiation pathways (Figure 3D). Future studies should directly test these mechanisms through LEF1 gain/loss‐of‐function experiments in T‐cell subsets.

It is noteworthy that this study also has many limitations. Our study proposes that LEF1 is a key gene which is upregulated in AAA and LEF1's association with T‐cell infiltration, but the function of LEF1 has not been demonstrated, and further in vitro and in vivo experiments are needed to explore the function of LEF1 in regulating T cells and thereby affecting aortic aneurysms. Especially, whether LEF1 knockdown/overexpression alters T‐cell cytokine profiles and whether T‐cell‐specific LEF1 deletion attenuates AAA progression in murine models remains to be investigated. In addition, the specific downstream mechanisms by which LEF1 functions in AAA remain to be further explored, which will be our major research endeavour in the future.

## 5 Conclusions

Our study identifies LEF1 as a potential biomarker for AAA, with elevated expression in T cells of human and murine AAA tissues. The correlation between LEF1 and immune infiltration (e.g., CD8^+^ T cells, macrophages) suggests its role in modulating inflammatory responses during AAA progression. However, the precise mechanisms by which LEF1 regulates T‐cell function in AAA—such as through Wnt/β‐catenin signalling or cytokine production—remain to be elucidated. Future studies should employ conditional LEF1 knockout models and in vitro assays to validate its causal role. These insights could pave the way for LEF1‐targeted therapies to mitigate immune‐mediated vascular remodelling in AAA.

## Author Contributions

**Pan He:** funding acquisition (equal), methodology (equal). **Xinyi Liu:** conceptualization (equal), resources (equal), visualization (equal). **Jiong Guo:** methodology (equal), software (equal). **Hanshen Luo:** conceptualization (equal), methodology (equal), writing – original draft (equal). **Yuehang Yang:** methodology (equal), validation (equal), writing – original draft (equal), writing – review and editing (equal).

## Conflicts of Interest

The authors declare no conflicts of interest.

## Supporting information


**Table S1:** jcmm70921‐sup‐0001‐TableS1.docx.

**Data S1:** jcmm70921‐sup‐0002‐DataS1.xls.

**Data S2:** jcmm70921‐sup‐0003‐DataS2.xls.

**Data S3:** jcmm70921‐sup‐0004‐DataS3.xls.

## Data Availability

The publicly available datasets analysed in this study (GSE47472, GSE57691, GSE7084 and GSE166676) can be found in the GEO database.
