# Supplementary material for: Identification LEF1 as a Potential Novel Biomarker for Abdominal Aortic Aneurysms Based on Comprehensive Bioinformatics Analysis
Source: J Cell Mol Med. 2025 Nov 9;29(21):e70921. doi: 10.1111/jcmm.70921 (PMC12597617; doi:10.1111/jcmm.70921)
Supplement: Supplementary file 1 — Table S1: jcmm70921‐sup‐0001‐TableS1.docx. [file JCMM-29-e70921-s004.docx]

**Table S1.** **Abdominal aortic aneurysm(AAA) datasets from several GEO datasets summarized.**

| **Series** | **Platform** | **GeneChip** | **Normal** | **AAA** | **Year** |
| --- | --- | --- | --- | --- | --- |
| GSE47472 | GPL10558 | Illumina HumanHT-12 V4.0 expression beadchip | 8 | 14 | 2013 |
| GSE57691 | GPL10558 | Illumina HumanHT-12 V4.0 expression beadchip | 10 | 49 | 2015 |
| GSE7084 | GPL2507 | Sentrix Human-6 Expression BeadChip | 8 | 7 | 2007 |
| GSE166676 | GPL24676 | Illumina NovaSeq 6000 (Homo sapiens) | 2 | 4 | 2021 |
